# Supplementary material for: The reaction-diffusion basis of animated patterns in eukaryotic flagella
Source: Nat Commun. 2023 Sep 27;14:5638. doi: 10.1038/s41467-023-40338-2 (PMC10533521; doi:10.1038/s41467-023-40338-2)
Supplement: Supplementary file 1 — Supplementary Information (SI) [file 41467_2023_40338_MOESM1_ESM.pdf]

The reaction-diffusion basis of animated patterns in eukaryotic flagella Supplementary Information (SI)

James F. Cass[1] and Hermes Bloomfield-Gadêlha[1]

[1]Department of Engineering Mathematics and Bristol Robotics Laboratory, University of Bristol, Bristol
hermes.gadelha@bristol.ac.uk

S1 Flagellar Chemo-ElastoHydrodynamic (chemoEH) Model Derivation

S1.1 Geometry

We consider a planar flagellum with position described by its inextensible centerline $\mathbf{r}(s,t)$ at point of arclength s and at time t. We idealize the axonemal structure as a pair of filaments $\mathbf{r}^+(s,t)$ and $\mathbf{r}^-(s,t)$, referred to as the filament bundle, of length L at a fixed separation $a \ll L$ measured in the normal direction across the centerline [1]. We will denote partial derivatives in space or time as a subscript s or t and often drop the explicit time and space dependence of quantities.

The tangent vector $\mathbf{t}(s) = \mathbf{r}_s(s)$ makes an angle $\theta(s)$ with the x-axis in the laboratory frame of reference $(\mathbf{x}, \mathbf{y})$, and we define the *shear angle* $\gamma(s) = \theta(s) - \theta(0)$ as the relative angle that the flagellum makes at point s with the basal end. Note that the unit tangent for the individual filaments $\mathbf{t}^{\pm}(s)$ is the same as for the centerline tangent $\mathbf{t}(s)$ when the spacing a is constant.

The normal vector $\mathbf{n}(s)$ is oriented as shown in Fig. 1 and we define the binormal vector as $\mathbf{b}(s) = \mathbf{t}(s) \times \mathbf{n}(s)$. The sliding displacment $\Delta(s)$ is the arclength separation between filaments at point s on the centerline so that with $\mathbf{r}^{\pm} = \mathbf{r} \pm (a/2)\mathbf{n}$ we have $\Delta(s) = \int_0^s (|\mathbf{r}_s^-| - |\mathbf{r}_s^+|) ds' = \Delta_0 + a\gamma(s)$ [1] where Δ_0 accounts for sliding at the basal end.

S1.2 Force and moment balance

Consider a small element of the structure of length ds as in Fig. S1, with active and passive cross-linkers grouped into a single central element, as in [2], exerting force per unit length $\mathbf{f} = f^t\mathbf{t} - f^n\mathbf{n}$ and moment per unit length $\mathbf{m} = m\mathbf{b}$. The reaction force f^n and moment m are required to satisfy the geometric constraint $\mathbf{r}^+ - \mathbf{r}^- = a\mathbf{n}$ that the filaments remain equally spaced. Equilibrium of moments about the centre of the internal element leads to $m = af^t/2$. Resultant internal forces $\mathbf{F}^{\pm} = T^{\pm}\mathbf{t} + N^{\pm}\mathbf{n}$ and moments $\mathbf{M}^{\pm} = M^{\pm}\mathbf{b}$ are exerted on the element by neighbouring material. The hydrodynamic force exerted by the external fluid on each filament (assumed equal) is denoted $\mathbf{h}^{\pm}$. Neglecting inertial forces due to the small Reynold's number, the balance of forces and moments across the element is given by

$$\mathbf{F}_s^+ + \mathbf{f} + \mathbf{h}^+ = 0 \tag{S.1}$$

$$\mathbf{F}_s^- - \mathbf{f} + \mathbf{h}^- = 0 \tag{S.2}$$

$$M_s^+ + N^+ + \frac{af^t}{2} = 0 \tag{S.3}$$

$$M_s^- + N^- + \frac{af^t}{2} = 0 \tag{S.4}$$

We model the elastic bending moment of the filaments with the linear Euler-Bernoulli relation $M^+ = M^- = B\theta_s/2$, where B is the bending rigidity of the bundle, neglecting the $O(a)$ difference in curvature between the filaments and centreline. Subtracting equation (S.4) from (S.3) gives $N^+ = N^-$, and hence equation (S.2) minus (S.1) gives the two scalar equations

$$\partial_s(T^+ - T^-) + 2f^t = 0 \implies T^+ - T^- = 2F^t \tag{S.5}$$

$$(T^+ - T^-)\theta_s - 2f^n = 0 \implies f^n = F^t\theta_s \tag{S.6}$$

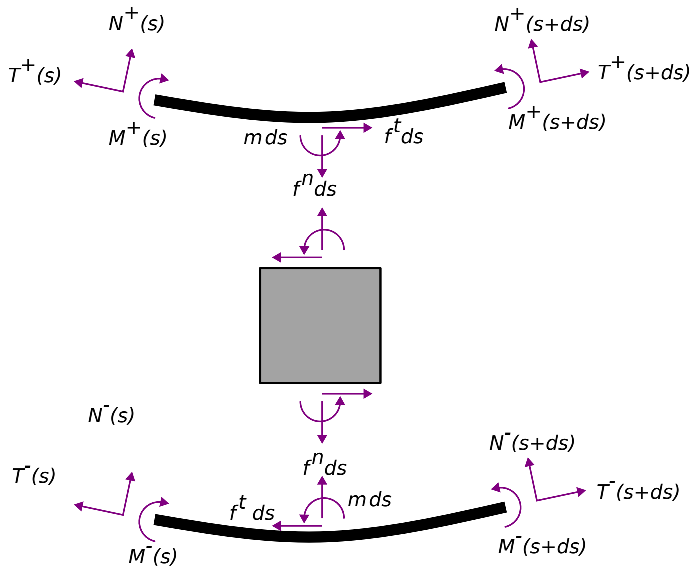

Figure S1: Free-body diagram of a small section of length ds of the axoneme, showing two filaments connected by a central element representing the combined active and passive cross-linking components exerting force per unit length $\mathbf{f} = f^t\mathbf{t} - f^n\mathbf{n}$ and moment per unit length $\mathbf{m} = m\mathbf{b}$.

where $F^t = \int_s^L f^t ds'$ is the integrated sliding force. Now summing the the pairs of equations (S.1)-(S.2) and (S.3)-(S.4) we obtain equations for the filament bundle

$$\mathbf{F}_s + \mathbf{h} = 0, \tag{S.7}$$

$$B\theta_{ss} + N + af^t = 0, \tag{S.8}$$

where $\mathbf{F} = \mathbf{F}^+ + \mathbf{F}^-$, $N = N^+ + N^-$ and $\mathbf{h} = \mathbf{h}^+ + \mathbf{h}^-$ are net quantities.

We use local resistive-force theory [3] to model the hydrodynamic force per unit length $\mathbf{h} = -(\xi^t\mathbf{tt} + \xi^n\mathbf{nn}) \cdot \mathbf{r}_t$. The fluid exerts anistropic drag forces resisting tangential and normal motion with drag coefficients ξ^t and ξ^n. We take $\xi^n = 4\pi\xi/\ln(L/a) = 2\xi^t$ for simplicity, where ξ is the fluid viscosity. Upon substitution equation (S.7) reads

$$\xi^n\mathbf{r}_t = 2(T_s - N\theta_s)\mathbf{t} + (T\theta_s + N_s)\mathbf{n}. \tag{S.9}$$

Differentiating this equation and applying the condition $\mathbf{r}_{st} \cdot \mathbf{r}_s = 0$, which enforces inextensibility of the centerline, leads to the scalar equations:

$$N_{ss} - 2N\theta_s^2 + 3T_s\theta_s + T\theta_{ss} - \xi^n\theta_t = 0, \tag{S.10}$$

$$2T_{ss} - T\theta_s^2 - 3N_s\theta_s - 2N\theta_{ss} = 0. \tag{S.11}$$

For a given f^t equations (S.8), (S.10)-(S.11) constitute a differential-algebraic system of partial differential equations for the unknowns $\theta(s,t)$, $N(s,t)$ and $T(s,t)$. Given solutions it is possible to construct the tangent vector $\mathbf{t} = (\cos\theta, \sin\theta)$ and finally the position vector $\mathbf{r} = \mathbf{r}_0 + \int_0^s \mathbf{t}ds'$, where $\mathbf{r}_0 = \mathbf{r}(s=0)$ can be calculated by integrating equation (S.9) evaluated at $s = 0$.

S1.3 The force f^t due to active and passive cross-linking elements

The tangential force f^t exerted between the two filaments consists of two elements. Firstly, there is an elastic resistance to shearing per unit length f^s which we model as a continuum of linear springs with spring constant K, so that $f^s = -K\Delta$. Secondly, molecular motors are assumed to lie along the filaments with constant density ρ, and the fraction of motors that are bound to the positive and negative filaments are labelled $n^+(s)$ and $n^-(s)$ (consistent with previous studies [4, 5]). We model the motor force per unit length f^m as a tug-of-war:

$$f^m = \rho(n^+F^- - n^-F^+), \tag{S.12}$$

where $F_\pm$ is the load exerted by a motor anchored on the plus or minus filament that is attempting to walk towards the base of the opposite filament (F^- tends to cause positive shearing). We model this load by a linear force-velocity relationship

$F_{\pm} = f_0(1 \pm \Delta_t/v_0)$, where f_0 is the force at stall ($\Delta_t = 0$) and v_0 is the velocity when the motors produce no force. The dyneins are thus force generators with internal damping elements associated with a constant drag coefficient $\xi^m = f_0/v_0$. Upon substitution the total tangential force becomes

$$f^t = f^m + f^s = \rho f_0 \left[\tilde{n} - \bar{n}\frac{\Delta_t}{v_0} \right] - K\Delta, \tag{S.13}$$

where $\bar{n} = n^- + n^+$ is the total proportion of bound motors at point s, and $\tilde{n} = n^- - n^+$ represents the differential binding of motors. Hence equation (S.13) contains a source term proportional to the differential binding $\tilde{n}$ and a dissipation term proportional to the total bound proportion of motors $\bar{n}$.

The dynamics of the bound motor populations n^+ follow

$$n_t^{\pm} = \pi_0(1 - n^{\pm}) - \epsilon_0 n^{\pm} \exp\left[\frac{f_0}{f_c}\left(1 \pm \frac{\Delta_t}{v_0}\right)\right], \tag{S.14}$$

as described in the main text. Equations (S.13) and (S.14) close the system (S.8), (S.10)-(S.11). Subject to suitable boundary and initial conditions this system can be solved numerically as described in Methods in the main text.

S1.4 Boundary conditions

We consider the distal end of the flagellum ($s = L$) to be free of forces and moments.

$$B\theta_s(L) = 0, \quad N(L) = 0, \quad T(L) = 0, \tag{S.15}$$

At the the basal end ($s = 0$), we consider a prolate spheroid head of major axis d and minor axis d' (see Fig. S2), rigidly connected to the flagellum so that the orientation of the head is the same as $\theta(0)$. The head dimensions were chosen to be $d = 4.5\mu$m and $d' = 2.8\mu$m, approximately the shape of a human sperm head [6]. Consistent with our use of local resistive force theory for the flagellum, we only consider contact forces between the body and the flagellum at $s = 0$ and ignore any effect of distant parts of the flagellum on the cell body through non-local hydrodynamic interactions.

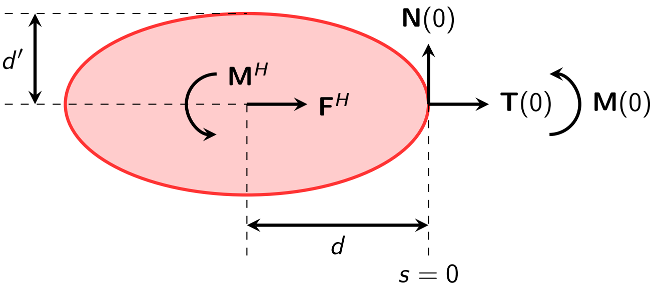

Figure S2: Balance of forces and moments on the cell body

Motion of the head is resisted by anistropic tangential and normal drag forces F^t, F^n so that $\mathbf{F}^H = F^t\mathbf{t}(0) + F^n\mathbf{n}(0)$ and a rotational drag $\mathbf{M}^H = M^H\mathbf{b}$. The translational and angular velocities of the head are related to these drag forces by [7]

$$F^t = -(6\pi\xi c_1 d)\tilde{v}^T, \tag{S.16}$$

$$F^n = -(6\pi\xi c_2 d)\tilde{v}^N, \tag{S.17}$$

$$M^H = -(8\pi\xi c_3 d(d')^2)\tilde{\Omega}. \tag{S.18}$$

where ξ is the fluid viscosity. The velocity $\tilde{\mathbf{v}} = \tilde{v}^T\mathbf{t}(0) + \tilde{v}^N\mathbf{n}(0)$ and angular velocity $\tilde{\Omega}$ relate to the center of mass of the

cell body. The constants c_i are functions of the eccentricity e of the spheroid [7],

$$c_1 = \frac{8e^3}{3\left[-2e + (1+e^2)\log\left(\frac{1+e}{1-e}\right)\right]}, \tag{S.19}$$

$$c_2 = \frac{16e^3}{3\left[2e + (3e^2-1)\log\left(\frac{1+e}{1-e}\right)\right]}, \tag{S.20}$$

$$c_3 = \frac{4e^3(2-e^2)}{3(1-e^2)\left[-2e + (1+e^2)\log\left(\frac{1+e}{1-e}\right)\right]}. \tag{S.21}$$

Balancing forces and torques on the cell body (see Fig. S2) at the point $s = 0$ leads to:

$$T(0) = 6\pi\xi c_1 d v^T, \tag{S.22}$$

$$N(0) = 6\pi\xi c_2 d(v^N - d\theta_t), \tag{S.23}$$

$$M(0) = \int_0^L N\,ds \tag{S.24}$$

$$= 8\pi\xi c_3 d(d')^2\theta_t(0) - dN(0). \tag{S.25}$$

where we have used the relationship $\tilde{\mathbf{v}} = \mathbf{v} - d\theta_t(0)\mathbf{n}$ between the velocity $\tilde{\mathbf{v}}$ of the center of mass and the velocity $\mathbf{v} = v^T\mathbf{t}(0) + v^N\mathbf{n}(0)$ of the point $s = 0$, which can be found by evaluating equation (S.9) at $s = 0$,

$$\xi_n v_t = 2(T_s - N\theta_s)|_{s=0}, \tag{S.26}$$

$$\xi_n v_n = (T\theta_s + N_s)|_{s=0}. \tag{S.27}$$

S1.5 Non-dimensionalisation

For numerical simulations, we non-dimensionalise s by the length of the flagellum L, time t by the motor time scale τ and all forces by the elastic bending force scale B/L^2. The complete closed system of equations and boundary conditions for the chemoEH flagellar model of a free-swimming sperm composed by a prolate spheroidal head is then

$$N_{ss} - 2N\theta_s^2 + 3T_s\theta_s + T\theta_{ss} - \mathrm{Sp}^4\theta_t = 0, \tag{S.28}$$

$$2T_{ss} - T\theta_s^2 - 3N_s\theta_s - 2N\theta_{ss} = 0, \tag{S.29}$$

$$\theta_{ss} + N + \mu_a(\tilde{n} - \zeta\bar{n}(\theta_t - \theta_t(0)) - \mu(\theta - \theta(0)) = 0 \tag{S.30}$$

$$n_t^\pm = \eta(1 - n^\pm) - (1-\eta)n^\pm e^{f^*(1\pm\zeta(\theta_t - \theta_t(0))} \tag{S.31}$$

$$\mathrm{Sp}^4 v^t = 2\left[T_s(0) - N(0)\theta_s(0)\right] \tag{S.32}$$

$$\mathrm{Sp}^4 v^n = T(0)\theta_s(0) + N_s(0) \tag{S.33}$$

$$N(0) = \frac{3}{2}\mathrm{Sp}^4\delta\log(\beta^{-1})c_2(v^t - \delta\theta_t), \tag{S.34}$$

$$T(0) = \frac{3}{2}\mathrm{Sp}^4\delta\log(\beta^{-1})c_1 v^n, \tag{S.35}$$

$$\int_0^1 N\,ds = 2\mathrm{Sp}^4\delta(\delta')^2\log(\beta^{-1})c_3\theta_t - \delta N, \tag{S.36}$$

where we have used the same symbols for the non-dimensional unknowns $(\theta, N, T, v^t, v^n, n^-, n^+)$ as for the dimensional quantities for simplicity of notation. See Table S1 for the forms of the non-dimensional parameters.

Parameter	Description	RD model	chemoEH model
$\mu = \frac{a^2 L^2 K}{B}$	shear resistance / bending resistance	10, 100	100
$\mu_a = \frac{a\rho f_0 L^2}{B}$	motor force / bending resistance	0.1-2.5, 2-18 ($\times 10^3$)	1-14 ($\times 10^3$)
$\zeta = \frac{a}{\tau v_0}$	flagellum diameter / motor distance	0.1-1	0.3
$\eta = \pi_0 \tau$	duty ratio	0.02 - 0.5	0.14
$f^* = \frac{f_0}{f_c}$	stall force / detachment force	2	2
$\mathrm{Sp} = [\xi_n/(B\tau)]^{\frac{1}{4}} L$	penetration length / flagellum length	-	1-13
$\beta = a/L$	flagellum diameter / flagellum length	-	0.003
$\delta = \frac{d}{L}$, $(\delta' = \frac{d'}{L})$	head major(minor) axis / flagellum length	-	0.077, 0.048*

Table S1: Non-dimensional parameters as ratios of characteristic forces/length scales. The motor time scale is $\tau = (\pi_0 + \epsilon_0)^{-1}$. *The minor axis ratio δ' is calculated from δ via $\delta' = \delta\sqrt{1-e^2}$ with fixed eccentricity $e = 0.782$. See equations (S.19)-(S.21) for the constants c_1, c_2, c_3.

S1.6 Numerical Methods

In order to simulate the chemoEH system above, we discretize spatially with second order finite differences and apply a differential-algebraic (DAE) equation solver, as described in Methods in the main text. The system of equations for the residuals that must be minimised at each time step reads

$$\mathrm{Sp}^4 v_t + n\,[3T_0 - 4T_1 + T_2 - N_1(3\theta_0 - 4\theta_1 + \theta - 2)] = 0 \tag{S.37}$$

$$\mathrm{Sp}^4 v_n + n\left[T_0\left(\frac{3}{2}\theta_0 - 2\theta_1 + \frac{1}{2}\theta_2\right) + \frac{3}{2}N_0 - 2N_1 + \frac{1}{2}N_3\right] = 0 \tag{S.38}$$

$$N_0 - \mathrm{Sp}_N^4(v_n - \delta\theta_{t,0}) = 0 \tag{S.39}$$

$$\mathrm{Sp}^4\theta_{t,i} - n^2\boldsymbol{\Delta}^2(N_i) + \frac{1}{2}n^2 N_i \boldsymbol{\Delta}(\theta_i)^2 - \frac{3}{4}n^2\boldsymbol{\Delta}(T_i)\boldsymbol{\Delta}(\theta_i) - n^2 T_i \boldsymbol{\Delta}^2(\theta_i) = 0 \tag{S.40}$$

$$N_{100} = 0 \tag{S.41}$$

$$\frac{1}{n}\left[\frac{1}{2}N_0 + N_1 + \dots + N_{99} + \frac{1}{2}N_{100}\right] - \mathrm{Sp}_\Omega^4\theta_{t,0} + \delta N_0 = 0 \tag{S.42}$$

$$N_i + n^2\boldsymbol{\Delta}^2(\theta_i) - \mu(\theta_i - \theta_0) + \mu_a\tilde{n}_i - \mu_a\zeta\bar{n}_i(\theta_{t,i} - \theta_{t,0}) = 0 \tag{S.43}$$

$$n\left[\frac{1}{2}\theta_{98} - 2\theta_{99} + \frac{3}{2}\theta_{100}\right] = 0 \tag{S.44}$$

$$T_0 - \mathrm{Sp}_T^4 v_t = 0 \tag{S.45}$$

$$2n^2\boldsymbol{\Delta}(T_i) - \frac{3}{4}n^2\boldsymbol{\Delta}(N_i)\boldsymbol{\Delta}(\theta_i) - 2n^2 N_i\boldsymbol{\Delta}^2(\theta_i) - \frac{1}{4}n^2 T_i\boldsymbol{\Delta}(\theta_i)^2 = 0 \tag{S.46}$$

$$T_{100} = 0 \tag{S.47}$$

where $\boldsymbol{\Delta}(\theta_i) = \theta_{i+1} - \theta_{i-1}$ and $\boldsymbol{\Delta}^2(\theta_i) = \theta_{i+1} - 2\theta_i + \theta_{i-1}$ are difference operators. Simulations were performed in the parameter space (Sp, μ_a) as given in Table S1.

S2 Flagellar Reaction-Diffusion (RD) Model

The reaction-diffusion model derives from considering the limit where hydrodynamics does not make any contribution to the internal balance of moments. From equation (S.7) we have that $N = \mathbf{n} \cdot \left(\int_s^L \mathbf{h}\, ds' \right)$ suggesting the correct scaling is $N \sim \xi_n L^2/\tau$ leading to the moment balance,

$$\frac{B}{L^2}\gamma_{ss} - a^2 K\gamma + a\rho f_0(\tilde{n} - \zeta\bar{n}\gamma_t) + \frac{\xi_n L^2}{\tau} N = 0, \tag{S.48}$$

where N is now $O(1)$ and non-dimensional, and we have made the changes $s \to s/L$, $t \to t/\tau$. Each of the coefficients in this equation have the units of a force. If the flagellum is oscillating with finite γ, and the parameter $\lambda = \xi_n L^2/(a^2 K\tau) \ll 1$, so that external viscous forces are small compared with internal elastic shearing forces [8], then the final term can be neglected in comparison with $a^2 K\gamma$. An alternative way to view the parameter λ is $(L/l_v)^2$, where $l_v = \sqrt{a^2 K\tau/\xi^n}$ is a length scale on which forces due to external viscosity are comparable with elastic shearing resistance. We relate viscous moments to external hydrodynamic dissipation by calculating

$$P^v = \mathbf{F}^H \cdot \tilde{\mathbf{v}} + M^H \tilde{\Omega} + \int_0^1 \mathbf{h} \cdot \mathbf{r}_t\, ds \tag{S.49}$$

The integral can be expanded using integration by parts

$$\int_0^1 \mathbf{h} \cdot \mathbf{r}_t\, ds = -\int_0^1 \mathbf{F}_s \cdot \mathbf{r}_t\, ds \tag{S.50}$$

$$= -[\mathbf{F} \cdot \mathbf{r}_t]_0^1 + \int_0^1 \mathbf{F} \cdot \mathbf{r}_{ts}\, ds \tag{S.51}$$

$$= \mathbf{F}(0) \cdot \mathbf{r}_t(0) + \int_0^1 N\theta_t\, ds \tag{S.52}$$

$$= \mathbf{F}(0) \cdot \mathbf{r}_t(0) + \mathbf{M}(0) \cdot \theta_t(0)\mathbf{k} + \int_0^1 N\gamma_t\, ds \tag{S.53}$$

Since $\mathbf{F}^H = -\mathbf{F}(0)$, $\mathbf{M}^H = -\mathbf{M}(0) - \delta N(0)\mathbf{k}$ and $\mathbf{v} = \mathbf{r}_t(0) - \delta\theta_t(0)\mathbf{n}(0)$ then the terms at $s = 0$ all cancel leaving

$$P^v = \int_0^1 N\gamma_t\, ds, \tag{S.54}$$

so that the hydrodynamic dissipation is proportional to the viscous moment per unit length N.

Neglecting this term and dividing through by B/L^2 we obtain the reduced system of partial differential equations, expressed as a generalised reaction-diffusion system

$$\mu_a\zeta\bar{n}\gamma_t = \gamma_{ss} - \mu\gamma + \mu_a\tilde{n}, \tag{S.55}$$

$$n_t^{\pm} = \eta(1 - n^{\pm}) - (1 - \eta)n^{\pm}e^{f^*(1 \pm \zeta\gamma_t)}, \tag{S.56}$$

for the unknowns (γ, n^-, n^+). Note that this system now describes the shape of the flagellum intrinsically through the shear angle γ rather than the lab frame tangent angle θ. To complete the system we must specify two boundary conditions (since the system is now only second order in s derivatives). In this limit, we are not concerned with the specific body coupling conditions, and only specify that there is no shearing permitted at the base, i.e. $\gamma(0) = 0$. At the distal end, we keep the condition of no external moment so that $\gamma_s(1) = 0$.

The system can be written in the form

$$\mathbf{M}(\mathbf{u})\mathbf{u}_t = \mathbf{D}\mathbf{u}_{ss} + \mathbf{L}\mathbf{u} + \mathbf{N}(\mathbf{u}, \mathbf{u}_t)$$

where

$$\mathbf{u} = \begin{bmatrix}\gamma & n_+ & n_-\end{bmatrix}^T \tag{S.57}$$

$$\mathbf{M}(\mathbf{u}) = \begin{bmatrix}\mu_a\zeta\bar{n} & 0 & 0\\ 0 & 1 & 0\\ 0 & 0 & 1\end{bmatrix} \tag{S.58}$$

$$\mathbf{D} = \begin{bmatrix}1 & 0 & 0\\ 0 & 0 & 0\\ 0 & 0 & 0\end{bmatrix} \tag{S.59}$$

$$\mathbf{L} = \begin{bmatrix}-\mu & \mu_a & -\mu_a\\ 0 & -\eta & 0\\ 0 & 0 & -\eta\end{bmatrix} \tag{S.60}$$

$$\mathbf{N}(\mathbf{u}, \mathbf{u}_t) = \begin{bmatrix}0\\ \eta - (1-\eta)n_+ e^{f^*(1+\zeta\gamma_t)}\\ \eta - (1-\eta)n_- + e^{f^*(1-\zeta\gamma_t)}\end{bmatrix}. \tag{S.61}$$

This form makes it clear that the system is a generalised reaction-diffusion system with a diagonal matrix $\mathbf{D}$ of diffusion coefficients and a state-dependent mass matrix $\mathbf{M}(\mathbf{u})$ (see Main Text). When $\mathbf{D} = 0$ the remaining terms only depend on local quantities.

S2.1 Isolated self-oscillating element

If the element in Fig. S1 is isolated from the surrounding flagellum, then there are no contact forces $\mathbf{F}$ and moments $\mathbf{M}$. Considering the small hydrodynamic limit as in the previous section, the internal cross-linking force per unit length $\mathbf{f}$ and moment $\mathbf{m}$ must vanish. Hence $f^t = 0$, which implies,

$$\rho f_0(\tilde{n} - \bar{n}\Delta_t/v_0) - K\Delta = 0 \tag{S.62}$$

The shear displacement Δ is now a scalar quantity, see Fig. 1 of the main text. Non-dimensionalising by length a and time τ gives the system of ordinary differential equations

$$\nu_a\zeta\bar{n}\gamma_t = -\gamma + \nu_a\tilde{n} \tag{S.63}$$

$$n_t^\pm = \eta(1-n^\pm) - (1-\eta)n^\pm e^{f^*(1\pm\zeta\gamma_t)}, \tag{S.64}$$

where $n^\pm$ are also now scalar quantities, $\gamma = \Delta/a$ is the shear angle and $\nu_a = \rho f_0/(aK)$ is the ratio of the characteristic motor force to the elastic restoring force. At a critical value of ν_a the system displays a Hopf bifurcation leading to growth from small pertubations about the straight equilibrium configuration to spontaneous shear oscillations, as in Fig. 2 of the main text.

S3 Linear Stability Analysis

The expressions obtained from the following linear stability analysis are more concise when the RD system is non-dimensionalised using the relaxation time scale $\bar{\tau} = (\pi_0 + \epsilon_0 e^{f^*})^{-1}$ of $n^\pm$. The non-dimensional RD system becomes

$$\mu_a\bar{n}\bar{\zeta}\gamma_t = \gamma_{ss} - \mu\gamma + \mu_a\tilde{n}, \tag{S.65}$$

$$n_t^\pm = n_0(1-n^\pm) - (1-n_0)n^\pm e^{\pm f^*\bar{\zeta}\gamma_t}, \tag{S.66}$$

where $\bar{\zeta} = a/(v_0\bar{\tau})$. There is an equilibrium solution when $(\gamma, n_\pm) = (0, n_0)$, where $n_0 = \pi_0\bar{\tau}$. Introducing a small perturbation around this solution so that $\gamma = \delta\gamma$ and $n_\pm = n_0 \pm \delta n$, we obtain the linearised system

$$2\mu_a\bar{\zeta}n_0\delta\gamma_t = \delta\gamma_{ss} - \mu\delta\gamma + 2\mu_a\delta n, \tag{S.67}$$

$$\delta n_t = -\delta n + (1-n_0)n_0 f^*\zeta\delta\gamma_t. \tag{S.68}$$

We attempt solutions of the form

$$\begin{bmatrix} \delta\gamma \\ \delta n \end{bmatrix} = \begin{bmatrix} A \\ B \end{bmatrix} e^{\sigma t} \sin(n + \tfrac{1}{2})\pi s + \text{c.c.}, \tag{S.69}$$

which satisfy the boundary conditions for arbitrary complex constants A, B and for integer n (c.c. indicates a complex conjugate). The complex eigenvalue $\sigma = \alpha + i\omega$ describes the growth rate and frequency of perturbations. When $\alpha = 0$ there is a Hopf bifurcation, with critical value μ_a^{crit} of the activity parameter μ_a and critical frequency ω_0. Solving with the ansatz (S.69) where we first set $\sigma = i\omega_0$ leads to

$$\omega_0^2 = (1 - n_0)f^* - 1, \tag{S.70}$$

$$\mu_a^{\text{crit}} = \frac{\pi^2 + 4\mu}{8\bar{\zeta} n_0 \omega_0^2} \tag{S.71}$$

To obtain the *smallest* value of μ_a^{crit} at which a spatial mode becomes unstable we have set $n = 0$. We then find the growth rate α and frequency ω near to the critical μ_a in terms of the relative distance from the bifurcation $\epsilon = (\mu_a - \mu_a^{\text{crit}})/\mu_a^{\text{crit}}$ and ω_0, obtaining

$$\alpha = \frac{\epsilon\omega_0^2}{2(1+\epsilon)}, \tag{S.72}$$

$$\omega^2 = \left[\frac{1 + \epsilon - \frac{1}{4}\omega_0^2\epsilon^2}{(1+\epsilon)^2}\right]\omega_0^2. \tag{S.73}$$

S4 Fitting with experimental data

We compared numerical simulations of the RD model with experimental data available from previous studies [9, 10] (see Results in main text). Table S2 summarizes the distinctions between the fitting procedure with nonlinear solutions used in this paper compared with previous linear fitting studies using the sliding-filament mechanism [5, 9].

**Feature**	**RD fitting**	**Bull sperm** (free swimming) [5]	**Chlamydomonas** (free-swimming isolated axonemes, wild-type/mbo2) [9]
governing equations	chemo-elastic (RD) geometrically nonlinear	elastic (hyperdiffusive) geometrically linear	elastic (hyperdiffusive) geometrically linear
molecular motor force f^m	tug-of-war (S.12)	linear geometric feedback $\tilde{f}^m = \chi\tilde{\Delta}$	linear geometric feedback $\tilde{f}^m = \mathrm{Re}(\chi)\tilde{\Delta} + \mathrm{Im}(\beta)\theta_s$
cross-linking reaction kinetics	yes (S.14)	no	no
data fitted with solutions obtained from	large-amplitude, nonlinear simulations far from equilibrium (total: 5460): $\gamma(s,t)$, $n^{\pm}(s,t)$	analytical solutions near the Hopf bifurcation: fundamental mode of the tangent angle $\tilde{\theta}(s)$	analytical solutions near the Hopf bifurcation: fundamental mode of the tangent angle $\tilde{\theta}(s)$
free parameters fitted	Total: 3<br>μ_a : active force<br>ζ : motor drag<br>η : duty ratio	Total: 7<br>$\mathrm{Re}(\chi)/\mathrm{Im}(\chi)$: linear elastic/viscous response to sliding<br>$\mathrm{Re}(\bar{\chi})/\mathrm{Im}(\bar{\chi})$: elastic/viscous basal sliding response<br>$\bar{\gamma}_p, \bar{\zeta}$: angular/translational drag on head<br>A : amplitude of $\tilde{\theta}$	Total: 5<br>$\mathrm{Re}(\chi)$: linear elastic response to sliding<br>$\mathrm{Im}(\beta)$: linear dynamic response to curvature<br>$\mathrm{Re}(\bar{\chi})/\mathrm{Im}(\bar{\chi})$: elastic/viscous basal sliding response<br>A : amplitude of $\tilde{\theta}$
Wave-amplitude fitting	no	yes	yes
fixed parameters (experimental estimates)	$\mu = a^2KL^2/B$: sliding/bending force	$\mathrm{Sp} = L(\xi^n\omega/B)^{\frac{1}{4}}$: hydrodynamic/bending force	$\mathrm{Sp} = L(\xi^n\omega/B)^{\frac{1}{4}}$: hydrodynamic/ bending force<br>C: static curvature
optimisation procedure	largest R^2	Maximize R^2 over free parameters	Maximize R^2 over free parameters
basal sliding present	no	yes	yes

Table S2: Features of the fitting procedure with the RD model (see Table 1 and Fig. 4 of the main text) compared with previous studies fitting sliding filament control [5, 9]. The comparison above is made only with the free swimming boundary condition for bull sperm [5], to contrast with our fitting for the free-swimming bull sperm data taken from [10]. The *Chlamydomonas* beating patterns were measured using isolated freely-swimming cilia [9]. Note that for the linear solutions for the fundamental mode $\tilde{\theta}_s$ the amplitude A and frequency ω are not determined parameters in the solution (free, fixed parameters respectively). They emerge naturally, however, from our RD nonlinear simulations after saturation of the nonlinear modes at advanced stages of the dynamics. Direct comparison between the fitting accuracy of the geometrically exact RD sliding-controlled model and linearised elastohydrodynamic sliding-control model in [9] for *Chlamydomonas* beating is provided in Fig. 6 of the main text.

As validation of our RD approximation, we also applied the nonlinear fitting procedure to contrast the beats produced by simulations of the chemoEH and RD models for the same parameter values (except Sp, only present for the chemoEH system). Fig. S3**a** shows that in the decoupling regime, the R^2 score is very close to 1, while Fig S3**b** shows the fraction of the power in the fundamental shape mode $\tilde{\gamma}_{\mathrm{sim}}(s)$ which is close to 1 in all cases. Combined, these plots indicate that the fundamental mode is a very accurate characterization of each simulation and that the fundamental mode of the RD simulation is a very accurate surrogate for the full chemoEH model.

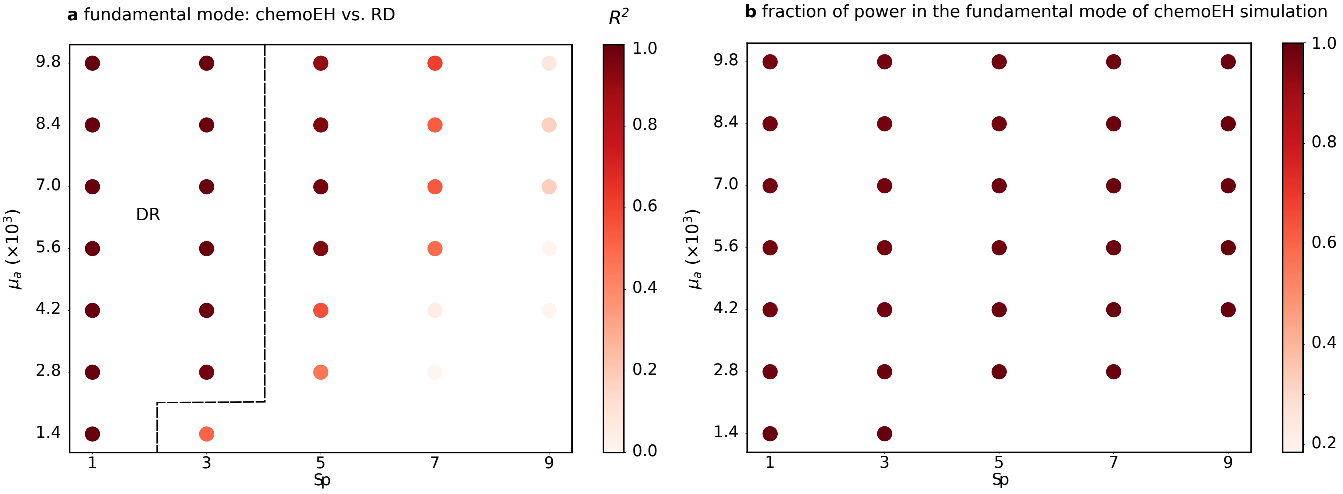

Figure S3: Accuracy of the reaction-diffusion approximation in the hydrodynamic decoupling region. **a** Closeness score $R^2(\tilde{\gamma}^{\mathrm{CEH}}_{\mathrm{sim}}, \tilde{\gamma}^{\mathrm{RD}}_{\mathrm{sim}})$. We apply the fitting procedure used in the main text to compare chemoEH simulations with the corresponding RD simulations, showing very high accuracy in the decoupling region (DR). **b** The fraction of power in the fundamental mode $\tilde{\gamma}_{\mathrm{sim}}$, at fundamental frequency ω_{sim}, of chemoEH simulations is consistently ≈ 1, as found in the experimental case [5]. Power is calculated as the modulus squared of the discrete Fourier transform (temporal) over many periods, averaged over arclength [5].

References

[1] Sébastien Camalet and Frank Jülicher. Generic aspects of axonemal beating. *New Journal of Physics*, 2(1):24, 2000.

[2] Philip V Bayly and Kate S Wilson. Equations of interdoublet separation during flagella motion reveal mechanisms of wave propagation and instability. *Biophysical journal*, 107(7):1756–1772, 2014.

[3] James Gray and GJ Hancock. The propulsion of sea-urchin spermatozoa. *Journal of Experimental Biology*, 32(4): 802–814, 1955.

[4] David Oriola, Hermes Gadêlha, and Jaume Casademunt. Nonlinear amplitude dynamics in flagellar beating. *Royal Society open science*, 4(3):160698, 2017.

[5] Ingmar H Riedel-Kruse, Andreas Hilfinger, Jonathon Howard, and Frank Jülicher. How molecular motors shape the flagellar beat. *HFSP journal*, 1(3):192–208, 2007.

[6] Hermes Gadêlha and Eamonn A Gaffney. Flagellar ultrastructure suppresses buckling instabilities and enables mammalian sperm navigation in high-viscosity media. *Journal of The Royal Society Interface*, 16(152):20180668, 2019.

[7] Allen T Chwang and TY Wu. Hydromechanics of low-reynolds-number flow. part 2. singularity method for stokes flows. *Journal of Fluid mechanics*, 67(4):787–815, 1975.

[8] Veikko F Geyer, Jonathon Howard, and Pablo Sartori. Ciliary beating patterns map onto a low-dimensional behavioural space. *Nature Physics*, pages 1–6, 2022.

[9] Pablo Sartori, Veikko F Geyer, Andre Scholich, Frank Jülicher, and Jonathon Howard. Dynamic curvature regulation accounts for the symmetric and asymmetric beats of chlamydomonas flagella. *Elife*, 5:e13258, 2016.

[10] Jeffrey S Guasto, Jonathan B Estrada, Filippo Menolascina, Lisa J Burton, Mohak Patel, Christian Franck, AE Hosoi, Richard K Zimmer, and Roman Stocker. Flagellar kinematics reveals the role of environment in shaping sperm motility. *Journal of the Royal Society Interface*, 17(170):20200525, 2020.
